# A patient with 46,XY/47,XYY karyotype and female phenotype: a case report

**DOI:** 10.1186/s12902-020-0523-8

**Published:** 2020-03-24

**Authors:** Zhi-Hui Liu, Shi-Chao Zhou, Jun-Wen Du, Kun Zhang, Tao Wu

**Affiliations:** Department of Endocrinology and Metabolism, The First Hospital of Shijiazhuang City, Shijiazhuang, Hebei China

**Keywords:** 47,XYY, Chromosome, Manifestation

## Abstract

**Background:**

47,XYY is a chromosomal abnormality syndrome that is typically observed in patients with a male phenotype. Few patients with XYY syndrome will have infertility. We here report a case of 46,XY/47,XYY syndrome diagnosed in a patient with a female phenotype.

**Case presentation:**

A 15-year-old patient with a female phenotype visited our hospital owing to a chief complaint of short stature as of the age of 6 years. She was diagnosed with dwarf syndrome at the age of 10, but no change was noted after 2 months of growth hormone treatment. The patient’s height was 136 cm and the weight was 29 kg, both of which were below the third percentile for her age/gender. In addition to short stature, the 4th and 5th metacarpals were short and there was no significant sex development. Karyotype analysis showed 47,XYY, and chromosomal microarray examination showed a chimera of 46,XY/47,XYY.

**Conclusion:**

This is an extremely rare case of 47,XYY abnormality in a patient with a female phenotype, with only one such known case reported previously. Since the cause is unknown, and symptoms of this syndrome are highly atypical and variable in childhood, clinicians should be aware of this possibility to avoid misdiagnosis and offer counseling and hormone therapy as needed to patients and their parents to improve their quality of life.

## Background

Forty-seven, XYY is a chromosomal abnormality syndrome that occurs in 1/1000 men [1, 2]. This abnormality is due to failure of the Y chromosome in separating during the meiosis stage II of spermiogenesis, so that some of the sperm contain two Y chromosomes [3]. Disease symptoms are atypical in childhood, children may be identified earlier if the practitioner is aware of the physical, medical, and neurodevelopmental features. Clues to the diagnosis of XYY include tall stature, macrocephaly, macroorchidism, hypotonia with normal genital development and actually enlarged testicles, hypertelorism, and tremor. But its clinical manifestations are diverse, such as asthma, attention deficit hyperactivity disorder and autism [4, 5]. This disease should be suspected if a patient presents with megeoma, magalocephaly, muscular hypotonia, muscle tremor, and behavioral or neurological abnormalities. Once diagnosed, the patient should also be screened for asthma and oral diseases. Although this disease is extremely rare in patients with a female phenotype, we here report such a case of 46,XY/47,XYY syndrome diagnosed in a patient at our center with a female phenotype.

## Case presentation

**Fig. 1:**
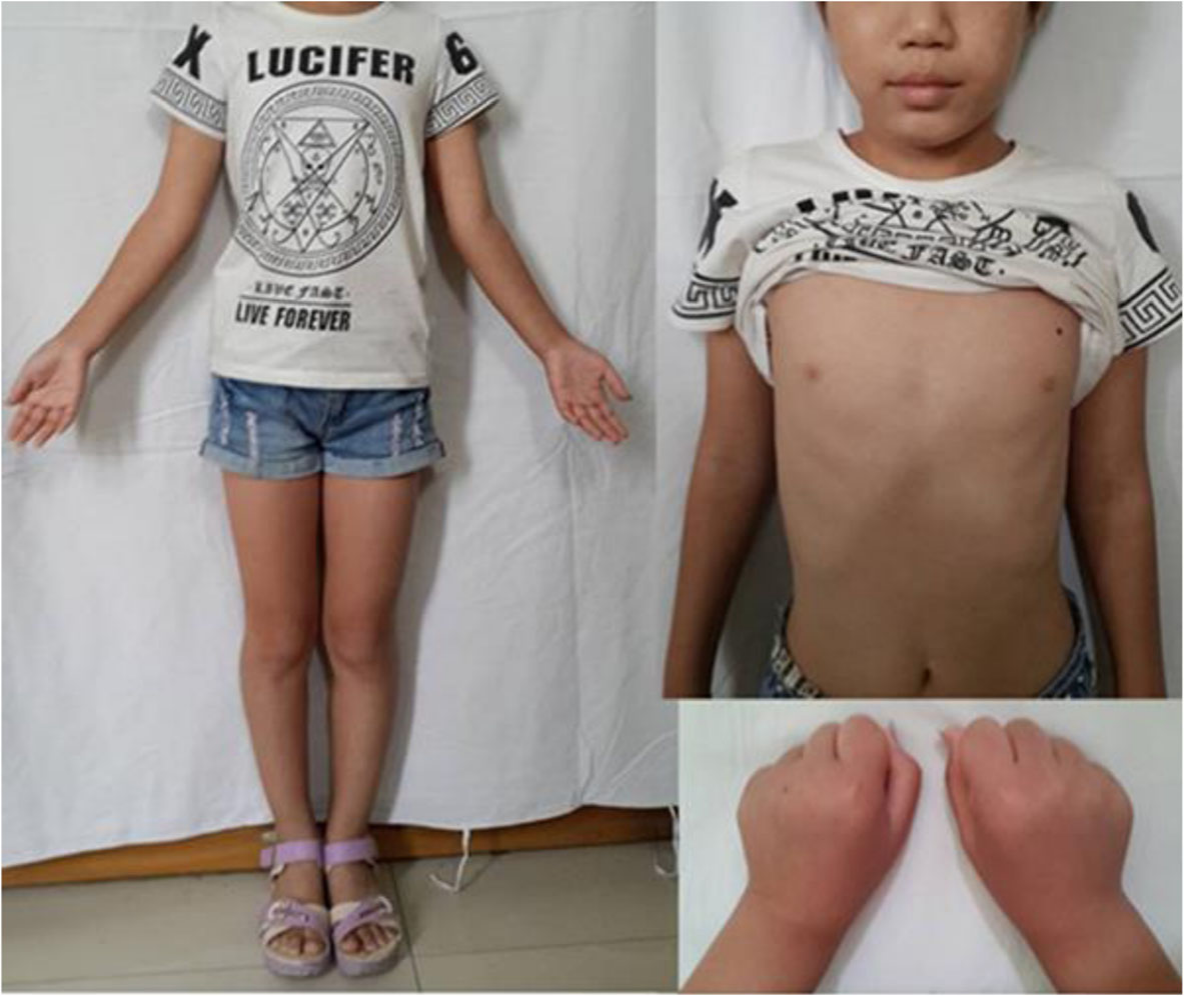
Characteristics of the patient

A 15-year-old patient with a female phenotype (Fig. 1) presented at our hospital with a chief complaint of short stature for 9 years. She was the first full-term child born to her parents; the mother was healthy during pregnancy and had no history of illness or medication. Her father was 167 cm in height; he began to develop at the age of 14 and had his first spermatorrhea at 18 years. Her mother was 157 cm in height, and her menarche began at the age of 12 years. Both parents stated no history of other hereditary and infectious diseases.

The patient was delivered via cesarean section, no postpartum asphyxia or hypoxia and no abnormal Apgar score. Her birth weight and length were 3.3 kg and 48 cm, respectively. She had normal teething, and mental and physical development before the age of 6 in line with her healthy peers. At the age of 6 years, she showed a slow growth rate, growing approximately 1–2 cm every year, with a gradual decline in intelligence, understanding, and learning ability. Five years ago, at the age of 10, her bone age was determined to be 8 years. The patient was then diagnosed with “dwarf syndrome” and treated with growth hormone. However, no height growth was obvious, and thus the treatment was terminated after 1–2 months.

Upon physical examination, the patient’s height was 136 cm and the weight was 29 kg, which are both below the third percentile of children of the same age and gender. The patient was lean, with a short neck, many moles, an epicanthic fold, tall palatine arches, a shield-like chest, wide breast distance, evident breath sounds in lungs, and no dry and wet rales. The heart rate was 86 beats per minute, with a regular rhythm. No murmur was heard in each valve auscultation area. The abdomen was soft, with no tenderness, rebound tenderness, and muscle tension, and there was no palpable swelling in the liver or spleen. Cubitus valgus was present. The 4th and 5th metacarpals were short, and no edema was detected in either of the lower limbs. In terms of sex development, she doesn’t have normal breast development. If the Tanner stage is adopted, the patient’s breast is stage I, which does not match the age. The patient had a young vulva and a small clitoris. There was no mass in the inguinal and labia majora area, and no pubic hair or armpit hair.

**Fig. 2:**
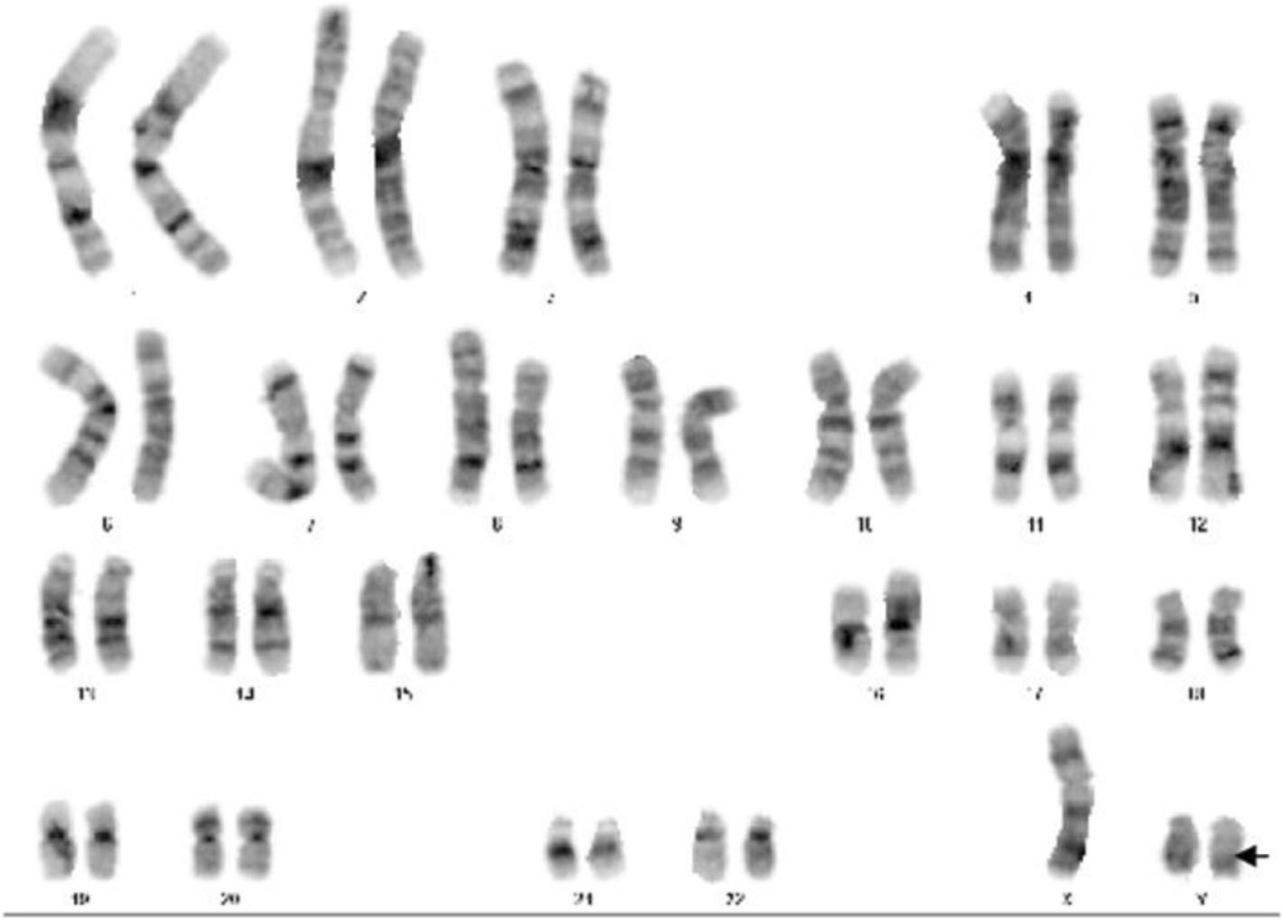
Chromosome karyotype analysis of the patient

Blood, urine, and stool analyses were unremarkable. Liver function, renal function, blood lipids, electrolytes, blood sugar, and thyroid function were all normal. Hepatitis tests revealed hepatitis A virus IgM antibody (−), hepatitis B surface antigen (−), hepatitis C virus antibody (−), and hepatitis E virus IgM antibody (−). Thyroid function and antibody were normal. Six sex hormones were analyzed: luteinizing hormone (LH), 17.5(0.1–11.9) mIU/ml; follicle-stimulating hormone (FSH), 103.50(2.1–11.1)mIU/ml; testosterone 0.09(0.00–2.39) nmol/l; progesterone, 1.50 (0.98–4.83)nmol/l; prolactin, 292.50 (6.68–53.44)μIU/ml, and estradiol < 18.35 (48.00–521.00)pmol/l, prolactin levels increased, but other biochemical and imaging tests were normal, which may be related to the patient’s nervousness, the above results suggest that the patient has hypergonadotrophic hypogonadism (gonadal failure/lack of functioning). Insulin-like growth factor-1 was 172.00 ng/ml. The 24-h urine free cortisol was normal. Karyotype analysis (400 belt, G belt) showed 47,XYY (Fig. 2). Further chromosomal microarray examination showed a chimera of the karyotype 46,XY/47,XYY. The ratio was approximately 2:1, and the Y chromosome contained the *SRY* gene.

**Fig. 3:**
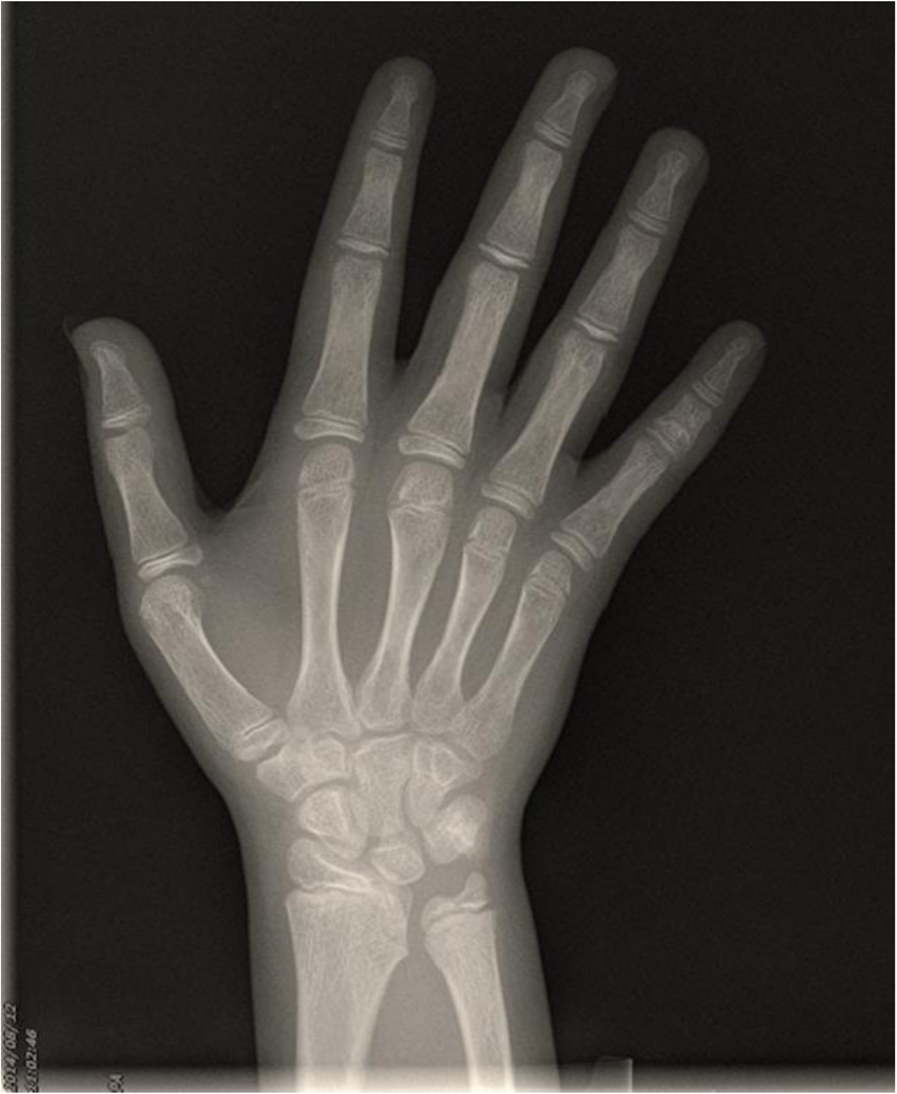
Bone age of the patient

Pituitary magnetic resonance imaging and chest X-rays showed no abnormalities. The bone age was approximately 13 years of age (Fig. 3). Ultrasound examination of the uterus and double attachment revealed an approximately 1.1 × 0.9 × 0.6-cm hypoecho, similar to a uterine echo. There was no endometrial echo and both ovaries were unclear. Cardiac ultrasonography showed no abnormalities in the intracardiac structure nor aortic stenosis. Abdominal color Doppler showed no abnormalities in the structures of the liver, gallbladder, pancreas, spleen, and kidneys.

Next-generation sequencing showed no mutations in the 65 genes associated with disorders of sex development, and no large missing or repeated segments of sex-reversal-related genes (*NROB1, WNT4, NR5A1, SOX9, AMH, SF1, FGF9, FOXL2*) were detected by multiplex ligation-dependent probe amplification.

## Discussion and conclusions

Chromosomal abnormalities are a common cause of male infertility, including the frequent infertility-related abnormality 47,XYY. Patients with this karyotype frequently have a chief complaint of behavioral abnormalities, increased adolescence growth rates, learning disabilities, and speech dysfunction. This shortened lifespan may be due to high-risk behaviors associated with the condition and severe trauma, or an increased risk of cancer, and respiratory and neurological diseases [3].

This disease should be suspected if a patient presents with megeoma, magalocephaly, muscular hypotonia, muscle tremor, and behavioral or neurological abnormalities. Thus, improvement of neurological assessments and electroencephalograms is required when necessary. FSH is particularly important for a diagnosis of primary gonadal dysgenesis, which has a longer half-life and is more sensitive and stable than LH [6].

Although the present patient’s karyotype analysis was 46,XY/47,XYY, the clinical phenotype is female. Maybe during the mitosis process, some cells have more Y chromosomes, and other cells have less Y chromosomes. Further research is needed on the number and morphology of more cells. So the clinical manifestations of patients have many similarities with Turner syndrome, maybe some cells have 45, XO. Or the fact that the chromosome Y is silent/suppressed, made this XY/XYY case look phenotypically as a XO, which may justify why some of the clinical manifestations are similar to Turner syndrome. Despite limited reports on this disease, one similar case was previously reported. A 14-year-old patient presented with short stature: the clinical phenotype was female, the chromosome karyotype was 47,XYY, there was no sign of Turner syndrome, and the mental and social abilities were normal [7]. The pathogenic mechanisms are currently unclear. This disease may be caused by genetic defects in certain signaling molecules in chromosome 45, XO/47, XXY, or androgen signaling pathways in certain tissues; alternatively, these symptoms may be due to the chromosomal abnormality. Unfortunately, owing to refusal of the family for further investigation, these speculations were not tested. In addition, although patients with 47,XYY are generally tall, the present patient has a short stature with a low IGF-1 level. Therefore, further growth hormone provocative tests and genetic screening may be helpful to pinpoint the cause. The physical, laboratory and imaging examinations all indicated primary gonadal dysgenesis. Although there is no pubertal development, the patient’s bone age reaches 13 years under the action of growth hormone, two years behind the actual age. However, regardless of the bone age or actual age, the patient’s height is far behind his peers. Furthermore, pelvic imaging examinations should be performed, and the immature gonads should be removed to avoid canceration. After excision of the gonadal tissue, gender selection should be carried out. Since the parents have been raising the patient as a girl thus far, it would be reasonable to select the female gender, and sex hormone replacement therapy should be applied simultaneously with growth hormones.

In conclusion, patients with a 47,XYY abnormality present with varying clinical manifestations; thus, diagnosis is generally delayed due to atypical features. Accordingly, clinicians should become aware of this possibility to make a timely diagnosis and improve the quality of life and options of such patients.

## Data Availability

All data generated and analyzed during this study are included in this published article and are available from the corresponding author upon reasonable request.

## Abbreviations

LH: Luteinizing hormone
FSH: Follicle-stimulating hormone
IGF-1: Insulin-like growth factor-1

## References

[1] Ratcliffe S (1999). Long-term outcome in children of sex chromosome abnormalities. Arch Dis Child.

[2] Borjian Boroujeni P, Sabbaghian M, Vosough Dizaj A, Zarei Moradi S, Almadani N, Mohammadpour Lashkari F (2019). Clinical aspects of infertile 47,XYY patients: a retrospective study. Hum Fertil (Camb).

[3] Kim IW, Khadilkar AC, Ko EY, Sabanegh ES (2013). 47,XYY syndrome and male infertility. Rev Urol.

[4] Zouli C, Tsametis C, Papadimas I, Goulis DG (2011). A man with 47, XYY karyotype, prolactinoma and a history of first trimester recurrent miscarriages in his wife. Hormones.

[5] Bardsley MZ, Kowal K, Levy C (2013). 47,XYY syndrome: clinical phenotype and timing of ascertainment. J Pediatr.

[6] El-Dahtory F, Elsheikha HM (2009). Male infertility related to an aberrant karyotype, 47,XYY: Four case reports. Cases J.

[7] Grace HJ, Campbell GD (1978). XYY karyotype, female phenotype and gonadal dysgenesis, A case report. S Afr Med J.

